# Supplementary material for: An optimized strategy to measure protein stability highlights differences between cold and hot unfolded states
Source: Nat Commun. 2017 May 18;8:15428. doi: 10.1038/ncomms15428 (PMC5454340; doi:10.1038/ncomms15428)
Supplement: Supplementary Information — Supplementary figures and supplementary references. [file ncomms15428-s1.pdf]

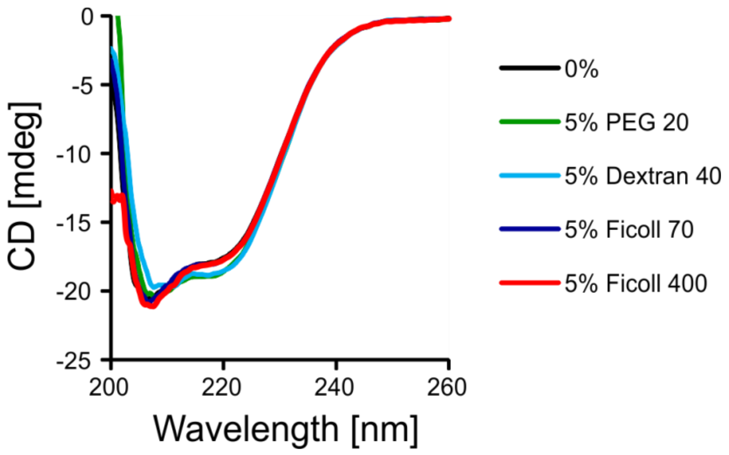

Supplementary Figure 1. Comparison of CD spectra. Superposition of the Far-UV CD spectrum of Yfh1 at 25 C in 20 mM HEPES buffer pH 7.0 (0%) with those in the presence, in the same buffer, of 5% of each of the crowders investigated. PEG 20 and Dextran 40 spectra are indistinguishable.

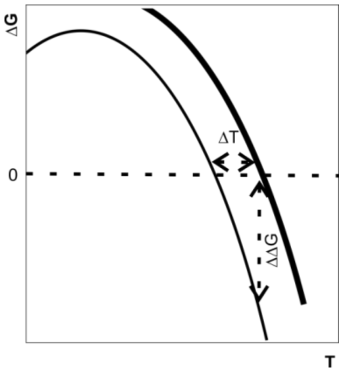

Supplementary Figure 2. **Relationship between ΔT and ΔΔG**.

As explained by Becktel and Schellman[1], the relationship between changes in melting temperature and changes in free energy is linear only if we can make the assumption that the portions of the two stability curves chosen to measure the temperature increment (*ΔT*) behave as parallel straight lines crossing the abscissa (*T*).

Figure adapted from Figure 8 of ref 1.

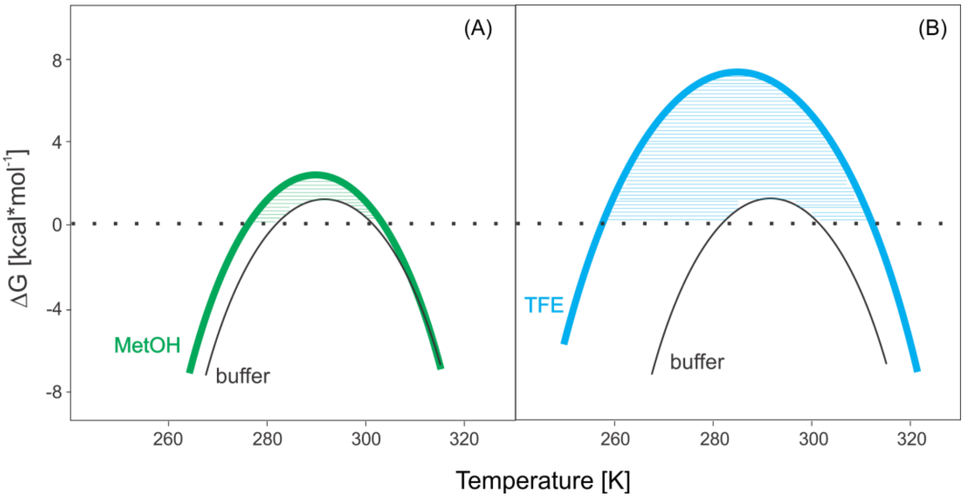

Supplementary Figure 3. **Variation of the stability curve of Yfh1 as a function of small concentrations of alcohols.** (A) increase of the area under the stability curve of Yfh1 after addition of 5% (v/v) methanol (black line for Yfh1 in buffer and green line for Yfh1 in MetOH). (B) increase of the area under the stability curve of Yfh1 after addition of 4% (v/v) trifluoroethanol (black line for Yfh1 in buffer and cyan line for Yfh1 in TFE). The dashed lines emphasize the increase of area under the stability curve. There is an increase in ΔG_S accompanied by small variations of T_m when studying the effect of low alcohol concentrations on Yeast frataxin[2]. The T_m point after addition of 5% methanol is essentially invariant within experimental error, but the ΔH_m value increases and the T_c point decreases, resulting in a large increase of the area under the curve. The changes after addition of 4% TFE are even more pronounced. We interpreted this behavior as a stabilization of the protein induced by alcohols, in spite of the widespread opinion that regards alcohols as typical denaturants.

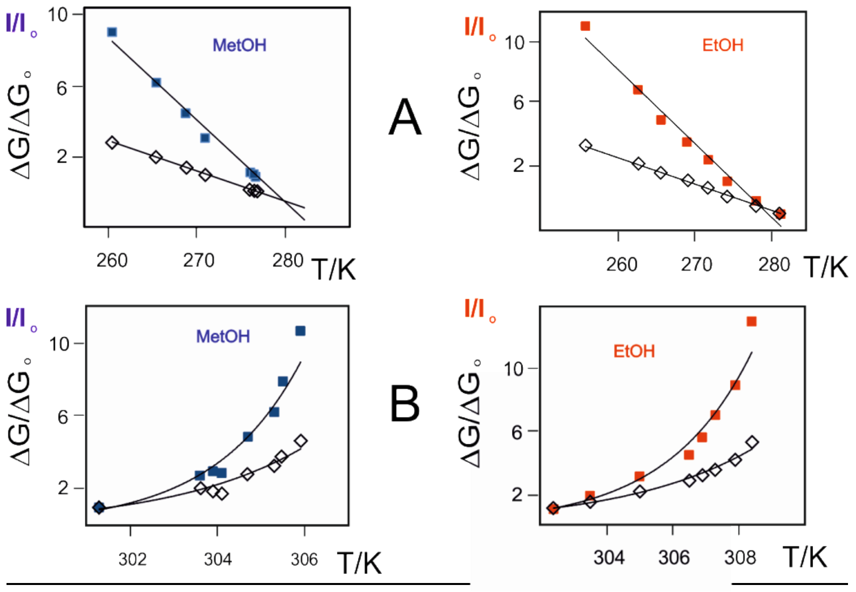

Supplementary Figure 4. Comparison of relative values of ΔG_S (hollow black diamonds) or I integrals (filled squares) of the stability curve of Yfh1 in several water-methanol and water-ethanol mixtures as a function of the temperature of unfolding. A) Dependence of ΔG_S or relative **I** integral values on the cold denaturation temperature upon addition of small amounts of methanol (from zero to 4%, v/v) or ethanol (from zero to 8%, v/v). B) Dependence of ΔG_S or relative **I** integral values on the heat denaturation temperature upon addition of small amounts of methanol (from zero to 4%, v/v) or ethanol (from zero to 8%, v/v). Upon addition of fairly small amounts of either methanol or ethanol, the relative values of the integrals increase linearly as the temperature of cold denaturation (T_c) decreases (Fig. 4 A), but increase exponentially with T_m. (Fig. 4 B). It is important to notice that in both cases the trend of relative integrals parallels that of the corresponding free energy change at maximum stability (ΔG_S). However, the changes in **I/I₀** are much larger. In fact, without the guide of the corresponding curves of **I/I₀** it would be difficult to spot an exponential increase in relative values of ΔG_S in the curves of Fig.4 B. The trends of the curves of relative integrals suggest intrinsically different mechanisms for heat and cold denaturation in the presence of alcohols.

Supplementary References

1. Becktel, W. J. & Schellman, J. A. Protein stability curves. *Biopolymers* **26**, 1859-77 (1987).
2. Martin, S. R., Esposito, V., De Los Rios, P., Pastore, A., & Temussi, P. A. Cold denaturation of yeast frataxin offers the clue to understand the effect of alcohols on protein stability. *J. Am. Chem. Soc.* **130**, 9963-70 (2008).
